# Triglyceride-glucose index: a novel prognostic marker for sepsis-associated encephalopathy severity and outcomes

**DOI:** 10.3389/fneur.2025.1468419

**Published:** 2025-04-02

**Authors:** Xiaopeng Shi, Lijun Xu, Jia Ren, Lijuan Jing, Xiangmei Zhao

**Affiliations:** ^1^Department of Emergency, Henan Provincial People’s Hospital, Zhengzhou, China; ^2^Zhengzhou University People’s Hospital, Zhengzhou, China

**Keywords:** sepsis-associated encephalopathy, triglyceride-glucose index, disease severity, prognosis, clinical outcomes

## Abstract

**Background:**

Sepsis-associated encephalopathy (SAE) is a complex condition with variable outcomes. This study investigates the potential of the Triglyceride-glucose (TyG) index as a marker for disease severity and prognosis in SAE patients.

**Methods:**

We conducted a retrospective cohort study using data from the Medical Information Mart for Intensive Care (MIMIC-IV) database. Patients with sepsis who were admitted to the intensive care unit (ICU) were categorized into two groups based on the occurrence of SAE. Key clinical outcomes were 90-day survival (primary outcome) and length of ICU and hospital stays, as well as the use of vasoactive medications (secondary outcomes). The TyG index was calculated, and its association with disease severity scores and patient outcomes was analyzed using statistical methods, including survival analysis, Cox regression, and correlation analyses.

**Results:**

The study population’s median age was 65.96 years, predominantly male (60.1%). Higher TyG index scores correlated with elevated clinical severity scores (APSIII, LODS, OASIS, SAPSII, and CCI) and increased ICU and hospital stay durations. TyG index categorization revealed significant differences in 90-day survival probabilities, with “high TyG” associated with a 25% increased mortality risk compared to “low TyG.” Furthermore, TyG index showed a moderate positive correlation with ICU stay duration and use of norepinephrine and vasopressin, but not with dopamine and epinephrine use.

**Conclusion:**

The TyG index is a significant independent predictor of disease severity and prognosis in SAE patients. High TyG levels correlate with worse clinical outcomes and increased mortality risk, suggesting its potential as a valuable tool in managing SAE.

## 1 Introduction

The global incidence of sepsis, a life-threatening complex disease, continues to escalate. Recent studies indicate that approximately 31 million individuals worldwide experience sepsis annually, with new cases in the United States ranging from 300 to 1,000 per 100,000 people (1). In China, the prevalence and mortality rates of sepsis surpass those observed in North America and Europe. A systematic review and meta-analysis estimate the incidence of sepsis in China to be 33.6% (2). Furthermore, the 90-day mortality rate for sepsis in Chinese intensive care units (ICUs) is reported at 35.5% (3). Sepsis-associated encephalopathy (SAE), a neurological dysfunction arising from severe infections and systemic inflammation, is linked to various factors, including inflammation, inadequate oxygenation, metabolic disorders, and drug effects (4). The clinical manifestations of SAE span from mild cognitive dysfunction to profound impairment of consciousness. The pathogenesis is intricate and multifactorial, encompassing vascular injury, endothelial activation, disruption of the blood–brain barrier, altered brain signaling, brain inflammation, and apoptosis (5). Extensive documentation affirms the independent association between SAE occurrence and short-term mortality. Even slight alterations in mental status independently elevate the risk of death (6), with the potential for enduring neurological sequelae (7). The evaluation of SAE in the ICU poses challenges and is presently conducted through clinical scoring systems (8). Notably, no specific biomarkers for SAE have been identified for use in routine clinical practice (9).

In recent years, the role of metabolic disorders in the pathophysiology of sepsis has received considerable attention (10). Studies have shown that hyperglycemia exacerbates blood–brain barrier (BBB) permeability and promotes neuronal apoptosis by activating the RAGE receptor pathway through advanced glycosylation end-products (AGEs) (11). Elevated triglyceride levels, on the other hand, result in the release of free fatty acids (FFAs), which activate microglial cells and trigger the release of pro-inflammatory cytokines such as IL-1β and TNF-*α*, initiating a neuroinflammatory cascade (12). Furthermore, insulin resistance exacerbates mitochondrial dysfunction and oxidative stress by inhibiting insulin signaling pathways in the brain, thereby impairing neuronal glucose uptake. These mechanisms suggest that metabolic markers may play a crucial role in the pathogenesis of sepsis-associated encephalopathy (SAE) (13). The triglyceride-glucose (TyG) index, as a surrogate marker of insulin resistance, has been widely used for risk prediction of cardiovascular disease and metabolic syndrome due to its simplicity and cost-effectiveness (14). Recent studies have shown that the TyG index is significantly associated with in-hospital mortality in sepsis patients (15), as well as with neurological outcomes such as microcirculatory disturbances and the risk of delirium (16). However, most existing studies have focused primarily on systemic sepsis or single-organ dysfunction, and research on the metabolic mechanisms underlying SAE, as well as its clinical assessment indices, remains insufficient.

This study investigated the TyG index as a potential predictor of severity and prognosis in SAE. The primary objective was to evaluate the correlation between TyG index and disease severity, survival, and the duration of ICU stay. Through the analysis of a substantial sample size and the application of reliable statistical methods, we aim to gain insights into the TyG index’s potential as a dependable marker for clinicians managing SAE. This endeavor is poised to contribute to the enhancement of care and prognosis for patients with SAE in the intensive care setting.

## 2 Materials and methods

### 2.1 Data source

This research leveraged the open-source medical information from the Medical Information Mart for Intensive Care (MIMIC-IV, version 2.2) database. MIMIC-IV is a comprehensive repository that encompasses patient data from Beth Israel Deaconess Medical Center, spanning the years 2008 to 2019 (17). This extensive database includes detailed medical records, medication regimens, laboratory results, patient demographics, and International Classification of Diseases (ICD) codes, offering a rich source of high-quality clinical data.

Team member Xiaopeng Shi has successfully completed the Collaborative Institutional Training Initiative (CITI) course offered by the National Institutes of Health (NIH) and obtained the requisite certification (Certification Number: 38652558). This certification, along with authorization from the Institutional Review Board (IRB) of the Massachusetts Institute of Technology (MIT), permits Xiaopeng Shi to access and utilize the MIMIC-IV database for research purposes.

Furthermore, this study received ethical approval from both the Massachusetts Institute of Technology (MIT IRB Number: 0403000206) and Beth Israel Deaconess Medical Center (BIDMC IRB Number: 2001-P-001699/14). Adhering to the highest standards of research integrity, the reporting of this study conforms to the Strengthening the Reporting of Observational Studies in Epidemiology (STROBE) guidelines (18).

### 2.2 Patients

#### 2.2.1 Inclusion criteria

(1) Alignment with the Sepsis 3.0 Definition and Diagnostic Criteria as delineated by the American Society of Critical Care Medicine and the European Society of Critical Care Medicine in 2016. This entails a diagnosis of Sepsis 3.0, characterized by an infection coupled with a Sequential Organ Failure Assessment (SOFA) score of 2 or higher. (2) Admission to the Intensive Care Unit (ICU) for treatment. For patients with multiple hospitalizations, the study only considered data from their initial ICU admission during the first hospitalization period. (3) Diagnosis of septic encephalopathy, defined by either a Glasgow Coma Scale (GCS) score of 14 or lower or a positive delirium assessment on the first day of ICU admission.

#### 2.2.2 Exclusion criteria

(1) Patients aged below 18 years. (2) Patients with an ICU stay of less than 24 h. (3) Patients presenting with primary brain injuries, which include traumatic brain injury, ischemic stroke, hemorrhagic stroke, epilepsy, intracranial infection, psychiatric disorders, or dementia. (4) Individuals with a history of chronic alcohol or drug abuse. (5) Absence of triglyceride or glucose laboratory results from the first day of ICU admission.

### 2.3 Study settings

In this study, patients with sepsis were categorized into two groups based on the occurrence of Sepsis-Associated Encephalopathy (SAE): the non-SAE group and the SAE group. The TyG index was calculated using the following formula: TyG = Ln[triglyceride (mg/dL) × glucose (mg/dL)/2]. This index is used to assess insulin resistance in patients and is a commonly used biomarker in sepsis research in recent years.

We assessed the following clinical outcomes:

Primary Outcome: Patient survival within the first 90 days following admission to the Intensive Care Unit (ICU).
Secondary Outcomes: (1) The total duration of the hospital stay. (2)The length of stay in the ICU. (3) Utilization of vasoactive medications, including epinephrine, norepinephrine, dopamine, and vasopressin.

### 2.4 Data collection

In this research, data extraction from the Medical Information Mart for Intensive Care (MIMIC-IV) database was executed using Structured Query Language (SQL) via PostgreSQL. Our focus was directed towards comprehensively gathering key data areas, as outlined below:

Demographic Information: This includes critical details such as the age and gender of the patients. Comorbidities: The presence of significant comorbidities was assessed, encompassing conditions like myocardial infarction, congestive heart failure, peripheral vascular disease, chronic lung disease, diabetes, chronic liver disease, and chronic kidney disease. Disease Severity Score at ICU Admission: We examined various scores indicative of disease severity on the first day of ICU admission. These included the Acute Physiology Score (APSIII), the Logistic Organ Dysfunction Score (LODS), the Oxford Acute Severity of Illness Score (OASIS), the Sequential Organ Failure Assessment (SOFA), and the Charlson Comorbidity Index (CCI). Vital Signs on ICU Admission Day: Vital signs recorded on the first day of ICU admission included heart rate (HR), respiratory rate (RR), mean arterial pressure (MAP), body temperature, and finger pulse oxygen saturation (SpO2). Laboratory Findings on ICU Admission Day: An array of laboratory parameters was collected, including hemoglobin, platelets, white blood cells, albumin, anion gap, bicarbonate, blood urea nitrogen, calcium, sodium, potassium, international normalized ratios (INR), prothrombin time (PT), partial thromboplastin time (PTT), alanine aminotransferase (ALT), aspartate aminotransferase (AST), total bilirubin (TBIL), blood oxygen saturation (SpO2), blood glucose, triglycerides, and lactate levels.

### 2.5 Statistical analysis

In this study, our initial step involved utilizing the Shapiro–Wilk test to evaluate the distribution of continuous variables. Based on the test outcomes, all continuous variables were determined to be non-normally distributed. Consequently, these variables were presented as medians and interquartile ranges (IQR). For the comparison between two groups, we employed the Mann–Whitney U test or the Wilcoxon rank sum test, depending on the data characteristics. Regarding categorical variables, these were expressed in terms of counts and percentages. The analysis of these variables was conducted using either the chi-square test (χ2 test) or the Fisher exact test, selected based on data suitability. Additionally, we employed Pearson’s correlation coefficient to measure the strength of association between the Triglyceride and Glucose index (TyG) and each scoring system. The relationships between these variables were visually depicted through heat maps for enhanced clarity.

For the analysis of factors influencing prognostic primary outcomes, survival analysis was conducted. This involved the use of the “surv_cutpoint” function in the R programming language to ascertain the optimal cut-off point, followed by the construction of Kaplan–Meier survival curves. The log-rank test was then applied to assess survival disparities between groups. Further analytical depth was added through both univariate and multivariate Cox regression analyses, with the findings presented via forest plots. To examine the correlations between TyG and secondary prognostic outcomes, Spearman rank correlation coefficients were utilized, and these relationships were visually represented through violin plots. All statistical analyses were performed using R sversion 4.3.2, and *p* < 0.05 was considered statistically significant.

## 3 Results

### 3.1 Baseline characteristics

**Figure 1:**
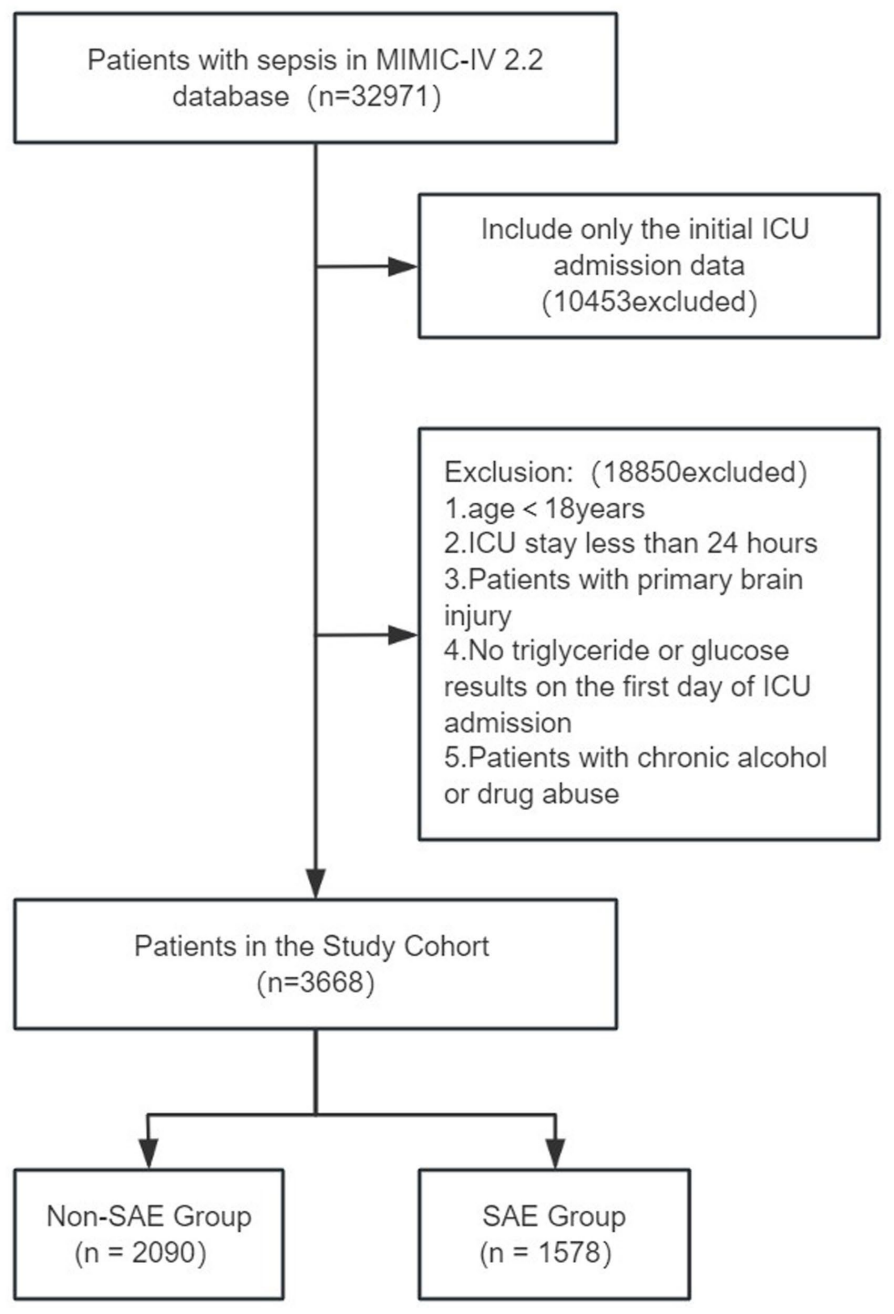
Flow chart for patient selection.

A total of 3,668 patients were enrolled in this study based on the predefined inclusion criteria, with 2090 patients in the non-SAE group and 1,578 patients in the SAE group. The patient recruitment process is outlined in Figure 1. Patients in the SAE group were significantly older than those in the non-SAE group (median age: 66.0 years vs. 64.0 years, *p* < 0.001). In terms of clinical scoring, the SAE group exhibited significantly higher scores for the Acute Physiological Score (APSIII), Organ Dysfunction Score (LODS), Oxford Acute Severity of Illness Score (OASIS), and Simplified Acute Physiology Score (SAPSII) (all *p* < 0.001), while no significant differences were observed between the two groups for the Sequential Organ Failure Assessment (SOFA) score (*p* = 0.202). Additionally, the Charlson Comorbidity Index (CCI) was significantly higher in the SAE group compared to the non-SAE group (*p* < 0.001).

Regarding vital signs, the mean arterial pressure (MAP), body temperature, and total bilirubin (TBIL) were significantly higher in the SAE group compared to the non-SAE group (all *p* ≤ 0.001). However, there were no significant differences between the groups in terms of heart rate, respiratory rate, or oxygen saturation (SpO2) (all *p* > 0.05). With respect to the incidence of comorbidities, no significant differences were found between the two groups in the rates of acute myocardial infarction (AMI), chronic obstructive pulmonary disease (COPD), or diabetes mellitus (DM) (all *p* > 0.05).

In terms of laboratory findings, the SAE group had significantly higher anion gap, blood urea nitrogen (BUN), sodium, prothrombin time (PT), and international normalized ratio (INR) compared to the non-SAE group (all *p* < 0.05). However, no significant differences were observed between the groups for blood lactate, platelet count, or white blood cell count. Notably, the triglyceride-glucose index (TyG) was significantly higher in the SAE group (*p* = 0.007) (Table 1).

**Table 1:** Baseline characteristics of the included patients.

Baseline variables	Total	Non-SAE group	SAE group	*p*-value
	*N* = 3,668	*N* = 2090	*N* = 1,578	
Age (years), median (Q1, Q3)	64.8 (53.9, 75.7)	64.0 (53.15, 74.85)	66.0 (54.9, 77.1)	<0.001
Male, *n* (%)	2,219 (60.5%)	1,271 (60.8%)	948 (60.1%)	0.658
Severe score, median (Q1, Q3)				
APSIII	53.0 (38.5, 67.5)	50.0 (37.0, 63.0)	58.0 (41.1, 74.9)	<0.001
LODS	6.0 (4.0, 8.0)	5.8 (4.45, 7.15)	7.0 (4.5, 9.5)	<0.001
OASIS	35.0 (29.0, 41.0)	33.0 (27.5, 38.5)	37.0 (31.0, 43.0)	<0.001
SAPSII	41.0 (31.5, 50.5)	39.0 (30.5, 47.5)	44.0 (33.1, 54.9)	<0.001
SOFA	3.0 (1.5, 4.5)	3.0 (1.5, 4.5)	3.0 (1.5, 4.5)	0.202
CCI	5.0 (3.0, 7.0)	5.0 (3.0, 7.0)	5.0 (3.0, 7.0)	<0.001
Vital signs, median (Q1, Q3)				
HR (beats/min)	88.9 (76.5, 101.3)	88.8 (76.6, 101.0)	89.5 (76.85, 102.15)	0.971
MAP (mmHg)	75.6 (69.35, 81.85)	75.3 (69.25, 81.35)	76.0 (69.55, 82.45)	0.001
RR (breath/min)	19.9 (16.95, 22.85)	19.9 (16.95, 22.85)	20.0 (17.0, 23.0)	0.565
Temperature (°C)	36.9 (36.5, 37.3)	36.80 (36.5, 37.1)	36.9 (36.55, 37.25)	<0.001
SPO2 (%)	97.2 (95.7, 98.7)	97.2 (95.65, 98.75)	97.1 (95.7, 98.5)	0.001
Comorbidity, *n* (%)				
AMI	745 (20.3%)	431 (20.6%)	314 (19.9%)	0.619
CHF	1,187 (32.4%)	661 (31.6%)	526 (33.3%)	0.285
PVD	434 (11.8%)	251 (12.0%)	183 (11.6%)	0.718
COPD	980 (26.7%)	561 (26.8%)	419 (26.6%)	0.851
CLD	835 (22.8%)	464 (22.2%)	371 (23.5%)	0.360
DM	1,140 (31.1%)	649 (31.1%)	491 (31.1%)	0.971
CKD	878 (23.9%)	517 (24.7%)	361 (22.9%)	0.197
Laboratory parameters, median (Q1, Q3)				
Hematocrit (%)	31.4 (26.85, 35.95)	31.1 (26.55, 35.65)	31.6 (26.9, 36.3)	0.343
Hemoglobin (g/dL)	10.3 (8.75, 11.85)	10.3 (8.75, 11.85)	10.4 (8.8, 12.0)	0.841
Platelets (K/uL)	183.0 (118.5, 247.5)	183.0 (119.5, 246.5)	183.0 (117.0, 249.0)	0.479
WBC (K/uL)	12.2 (8.35, 16.05)	12.0 (8.3, 15.7)	12.4 (8.25, 16.55)	0.137
Anion gap (mEq/L)	15.0 (12.5, 17.5)	15.0 (12.5, 17.5)	15.0 (12.75, 17.25)	0.008
Bicarbonate (mEq/L)	22.0 (19.0, 25.0)	22.0 (19.25, 24.75)	22.0 (19.0, 25.0)	0.262
BUN (mg/dL)	24.0 (11.45, 36.55)	23.5 (11.25, 35.75)	24.5 (11.5, 37.5)	0.014
Calcium (mg/dL)	8.2 (7.2, 9.2)	8.2 (7.3, 9.1)	8.2 (7.2, 9.2)	0.180
Chloride (mg/dL)	104.0 (96.0, 112.0)	104.0 (96.0, 112.0)	104.0 (96.0, 112.0)	0.868
Creatinine (mg/dL)	1.1 (0.1, 2.2)	1.2 (0.1, 2.3)	1.1 (0.1, 2.2)	0.342
Sodium (mEq/L)	138.5 (133.0, 144.0)	138.0 (133.0, 143.0)	138.5 (132.5, 144.5)	0.008
Potassium (mEq/L)	4.2 (3.4, 5.0)	4.2 (3.4, 5.0)	4.2 (3.3, 5.1)	0.972
Lymphocytes (K/uL)	1.2 (0.72, 1.91)	1.30 (0.76, 1.30)	1.30 (0.82, 1.36)	0.379
Monocytes (K/uL)	0.78 (0.43, 0.78)	0.76 (0.45, 0.78)	0.79 (0.46, 0.78)	0.409
Neutrophils (K/uL)	11.6 (5.8, 19.0)	11.5 (5.7, 18.7)	11.9 (5.6, 19.4)	0.193
INR	1.3 (0.9, 1.7)	1.3 (0.9, 1.7)	1.3 (0.8, 1.8)	<0.001
PT (sec)	14.4 (9.5, 19.3)	14.4 (9.9, 18.9)	14.5 (9.1, 19.9)	<0.001
PTT (sec)	32.4 (14.8, 49.6)	32.7 (14.3, 51.1)	32.0 (15.3, 48.7)	0.504
ALT (U/L)	23 (0, 59)	23 (0, 58.38)	22.5 (7, 59.5)	0.367
ALP (U/L)	67 (0, 104)	65 (0, 102)	68.5 (28, 107.38)	0.22
AST (U/L)	35.5 (6, 94)	34 (0, 91)	37 (13, 97.75)	0.404
TBIL (mg/dL)	0.55 (0, 1.4)	0.5 (0, 1.3)	0.6 (0.2, 1.45)	0.001
CK (U/L)	0 (0, 153.12)	0 (0, 158)	0 (0, 149.75)	0.355
CKMB (ng/mL)	0 (0, 4)	0 (0, 4)	0 (0, 4)	0.95
LDH (U/L)	0 (0, 336.25)	0 (0, 321.5)	143 (0, 353.88)	0.057
Lactate (mmol/L)	1.7 (1, 2.75)	1.67 (0.95, 2.8)	1.7 (1, 2.7)	0.521
PO2 (mmHg)	105.5 (62, 182.5)	111.5 (65, 194)	100 (59.5, 167)	<0.001
PCO2 (mmHg)	40 (33, 45.5)	39.5 (33, 45)	40 (33, 46)	0.146
Triglyceride (mg/dl)	128 (88, 199)	129 (88, 201)	128 (87, 198)	0.147
Glucose (mg/dl)	136 (112.5, 175)	132 (112.62, 179.38)	136 (113.58, 185.26)	0.019
TyG	9.18 (8.65, 9.85)	9.12 (8.65, 9.69)	9.26 (8.64, 9.82)	0.007
Outcomes				
los_hospital, median (Q1, Q3)	14.89 (8.82, 24.93)	14.9 (8.84, 25.21)	14.88 (8.78, 24.56)	0.949
los_icu, median (Q1, Q3)	5.95 (3.36, 11.73)	5.88 (3.36, 11.66)	6.04 (3.38, 11.88)	0.612
Dopamine, *n* (%)	207 (5.6%)	132 (6.3%)	75 (4.1%)	0.002
Epinephrine, *n* (%)	300 (8.1%)	189 (9.0%)	111 (7.0%)	0.028
Norepinephrine, *n* (%)	1708 (46.5%)	953 (45.5%)	756 (47.9%)	0.167
Vasopressin, *n* (%)	734 (20.4%)	396 (18.9%)	338 (21.4%)	0.068
Survival, *n* (%)	936 (25.5%)	445 (28.2%)	491 (23.5%)	0.001

Categorical data are presented as frequencies (percentages), and parametric continuous data are presented as medians (interquartile ranges). APSIII, Acute Physiology Score III; LODS, Logistic Organ Dysfunction System; OASIS, Oxford Acute Severity of Illness Score; SAPSII, Sequential Organ Failure Assessment (SOFA) Score; SOFA, Sequential Organ Failure Assessment; CCI, Charlson Comorbidity Index; AMI, Acute Myocardial Infarction; CHF, Congestive Heart Failure; PVD, Peripheral Vascular Disease; COPD, Chronic Obstructive Pulmonary Diseases; CLD, Chronic Liver Disease; DM, Diabetes Mellitus; CKD, Chronic Kidney Disease; HR, Heart Rate; RR, Respiratory Rate; MAP, Mean Arterial Pressure; BUN, Blood Urea Nitrogen; WBC, White Blood Cell count; INR, International Normalized Ratio; PT, Prothrombin Time; PTT, Partial Thromboplastin Time; ALT, Alanine Aminotransferase; AST, Aspartate Aminotransferase; TBIL, Total Bilirubin; NLR, Neutrophil to Lymphocyte Ratio; TyG, Triglyceride-Glucose Index.

### 3.2 Correlation between TyG index and disease severity scores

In SAE patients, we analyzed the correlation between the TyG index and Disease Severity Scores. The strongest correlation was found between the TyG index and the APSIII score, with a Pearson correlation coefficient of 0.1347602. However, this correlation is relatively weak, suggesting a loose linear relationship between the TyG index and the APSIII score. When compared to other scoring systems (such as LODS, OASIS, and SAPSII), the correlation between the TyG index and these systems also shows a weak positive association.

**Figure 2:**
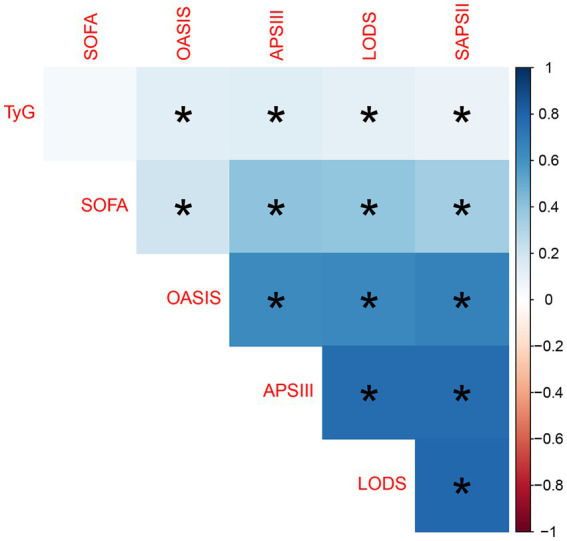
Correlation heatmap between TyG index and various disease severity scores. A darker blue color indicates a positive correlation, while a darker red indicates a negative correlation. The presence of an asterisk (*) denotes statistical significance, where a *p*-value is less than 0.05, indicating a reliable association between the variables.

Regarding the correlations between the scoring systems, strong positive correlations were found among the APSIII, LODS, and SAPSII scores, with Pearson correlation coefficients greater than 0.7, suggesting that these scoring systems may exhibit similar trends in assessing disease severity. In contrast, the correlation of SOFA with other scoring systems was relatively weak, particularly with the OASIS score (Pearson correlation coefficient = 0.20297439), indicating that the SOFA score may measure different aspects of disease severity, potentially reflecting different clinical dimensions. This distinction underscores the complexity and multifaceted nature of severity assessment in SAE patients, highlighting the necessity of a multifactorial approach in evaluating patient conditions (Figure 2).

### 3.3 Correlation between TyG and prognostic primary outcomes in SAE patients

**Figure 3:**
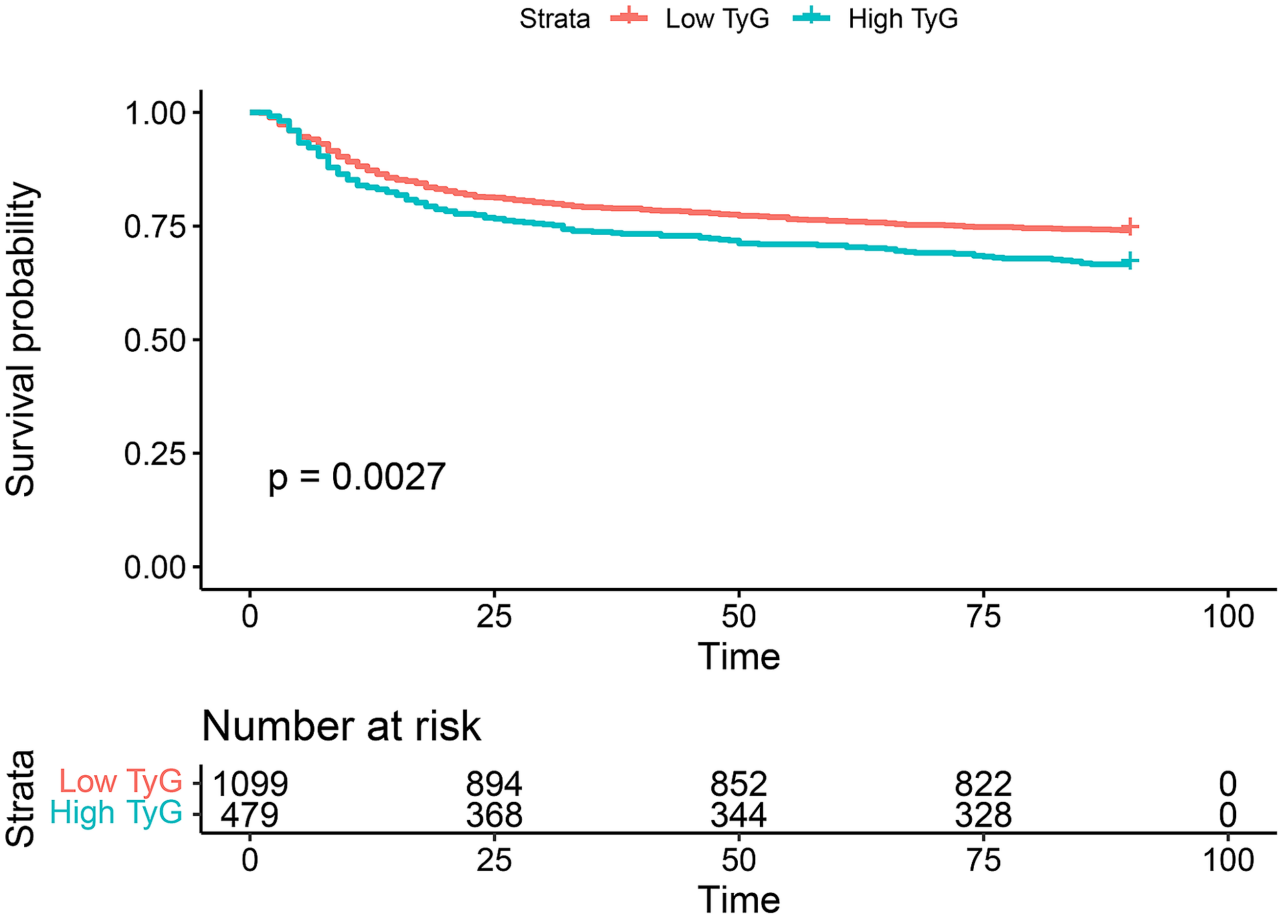
90-day Kaplan–Meier survival comparison between the low TyG index group and high TyG index group. “Low TyG” refers to individuals with a TyG index below 8.7639, whereas “High TyG” denotes individuals with a TyG index of 8.7639 or higher. Survival differences between the groups are assessed using the Log-rank test, with a *p*-value of less than 0.05 indicating statistical significance.

In SAE, we identified the optimal cutoff value for the TyG index as 8.7639. Using this cutoff, the TyG index was categorized into two groups: “Low TyG” and “High TyG.” Analysis of Kaplan–Meier survival curves (as depicted in Figure 3) revealed a temporal divergence in survival probabilities between these groups. Notably, the “low TyG” group exhibited a significantly higher survival probability compared to the “high TyG” group, as substantiated by a *p*-value of 0.0027 (Figure 3).

Table 2 elucidates that the univariate analysis has revealed an extensive array of both clinical and laboratory determinants significantly influencing the 90-day survival rate of patients with septic encephalopathy. These determinants span metabolic indicators such as the TyG index and age, a variety of scores assessing critical illness, blood constituents like hematocrit and hemoglobin, electrolyte levels, and vital physiological metrics including heart rate, mean arterial pressure, respiratory rate, and body temperature. Subsequent multivariate Cox regression analysis has pinpointed a select group of these factors as independent prognostic indicators, encompassing metabolic rates, specific illness severity scores, coexisting conditions such as congestive heart failure and chronic liver disease, alongside particular hematological and physiological parameters. The univariate regression analysis demonstrates that the TyG index is a significant predictor of mortality, with a hazard ratio (HR) of 1.344 and a *p*-value of 0.00275, underscoring a strong statistical significance. Upon adjustment for confounding factors in the multivariate analysis, the TyG index retains its prognostic value (*p* = 0.036114), with an HR of 1.2517. This outcome suggests that individuals with higher TyG levels have a 25% greater risk of mortality compared to those with lower levels, even when other variables are taken into account.(Talbe2).

**Table 2:** Univariate and multifactorial analyses of 90-day survival in patients with septic encephalopathy.

	Univariate analysis				Multivariate analysis				
			95%CI				95%CI		
Data	*p*	HR	Lower	Upper	*p*	HR	Lower		Upper
TyG (low, high)	0.00275	1.344	1.108	1.631	0.031614	1.2517	1.02		1.5360
Age	<0.01	1.024	1.017	1.03	0.001619	1.015743	1.0059	1.0257	
Male	0.224	0.8895	0.7366	1.074					
APSIII	<0.01	1.019	1.016	1.022	0.048133	1.0048	0.9914		1.0184
LODS	<0.01	1.129	1.101	1.159	0.813238	0.9932	0.9384		1.0512
OASIS	<0.01	1.039	1.028	1.05	0.25813	1.0101	0.9927	1.0279	1.0278
SAPSII	<0.01	1.031	1.026	1.036	0.482502	1.0048	0.9914		1.0184
SOFA	<0.01	1.128	1.094	1.163	0.023656	1.0496	1.0065		1.0946
CCI	<0.01	1.173	1.14	1.206	<0.01	1.1193	1.0732		1.1673
AMI	0.746	1.039	0.823	1.312					
CHF	0.0201	1.255	1.036	1.521	0.040457	0.7882	0.6278		0.9897
PVD	0.0601	1.291	0.9892	1.686					
COPD	0.101	1.186	0.9674	1.453					
CLD	<0.01	1.687	1.382	2.059	0.239308	1.1719	0.8998		1.5263
DM	0.405	0.9172	0.7484	1.124					
CKD	0.0609	1.225	0.9908	1.515					
Hematocrit	<0.01	0.9677	0.9536	0.9819	0.395879	1.0272	0.9655		1.0928
Hemoglobin	<0.01	0.885	0.8467	0.925	0.282313	0.9032	0.7502		1.0874
Platelets	0.04	0.9989	0.9979	1	0.269298	0.9995	0.9986		1.0004
WBC	<0.01	1.013	1.007	1.02	0.048444	0.9863	0.9728		0.9999
Aniongap	<0.01	1.06	1.041	1.078	0.086362	0.9578	0.9117		1.0062
Bicarbonate	<0.01	0.9602	0.9413	0.9796	0.708646	0.9908	0.9438		1.0401
BUN	<0.01	1.012	1.009	1.015	0.025329	1.0055	1.0007		1.0103
Calcium	0.131	1.084	0.9763	1.203					
Chloride	<0.01	0.9727	0.9603	0.9851	0.065698	0.9574	0.9141		1.0028
Creatinine	<0.01	1.1	1.053	1.149	0.060733	0.9139	0.8319		1.0041
Sodium	0.00357	0.9761	0.9603	0.9921	0.29484	1.0258	0.9781		1.0758
Potassium	<0.01	1.301	1.146	1.478	0.178602	1.1059	0.955		1.2806
Lymphocytes	0.0579	0.8858	0.7815	1.004					
Monocytes	0.891	1.004	0.9499	1.061					
Neutrophils	<0.01	1.025	1.017	1.034	0.000133	1.0354	1.0171		1.054
INR	<0.01	1.243	1.173	1.316	0.794342	0.9216	0.498		1.7028
PT	<0.01	1.024	1.018	1.03	0.60731	1.0162	0.9557		1.0806
PTT	<0.01	1.013	1.009	1.017	0.004401	1.007	1.0022		1.0118
ALT	0.0502	0.9998	0.9996	1					
ALP	<0.01	1.002	1.001	1.002	0.002041	1.0013	1.0005		1.0021
AST	0.132	0.9999	0.9998	1					
TBIL	<0.01	1.03	1.021	1.04	0.099284	1.0121	0.9977		1.0267
CK	0.35	1.01	0.999	1.02					
CKMB	0.173	1.001	0.9993	1.004					
LDH	0.811	1.001	0.998	1.002					
Lactate	<0.01	1.108	1.072	1.146	0.260307	1.0353	0.9746		1.0997
PO2	<0.01	0.9956	0.9942	0.9971	0.000261	0.9967	0.9949		0.9985
PCO2	0.279	0.9948	0.9855	1.004					
HR	0.00452	1.008	1.002	1.013	0.000708	1.0111	1.0047		1.0176
MAP	<0.01	0.9689	0.9593	0.9786	0.006051	0.9849	0.9743		0.9957
RR	<0.01	1.044	1.022	1.065	0.000708	1.0331	1.0074		1.0594
Temperature	<0.01	0.6412	0.5632	0.73	0.001289	0.7563	0.638		0.8965
SPO2	0.0245	0.9545	0.9165	0.994	0.63719	1.0121	0.9629		1.0638

HR, hazard ratio; 95%CI, 95% confidence interval, *p*-value < 0.05 denotes that the results are statistically significant.

Age also emerged as a significant predictor in the multivariate analysis. Each additional year of age was associated with a 1.6% increase in the risk of death (HR = 1.015743, *p* = 0.001619), a finding that remained robust even after controlling for other factors. Notably, the significance of certain variables, such as APSIII, SOFA, and CCI, diminished in the multivariate analysis. This attenuation could be attributed to their covariance with other variables or the adjustment effects of the latter.

**Figure 4:**
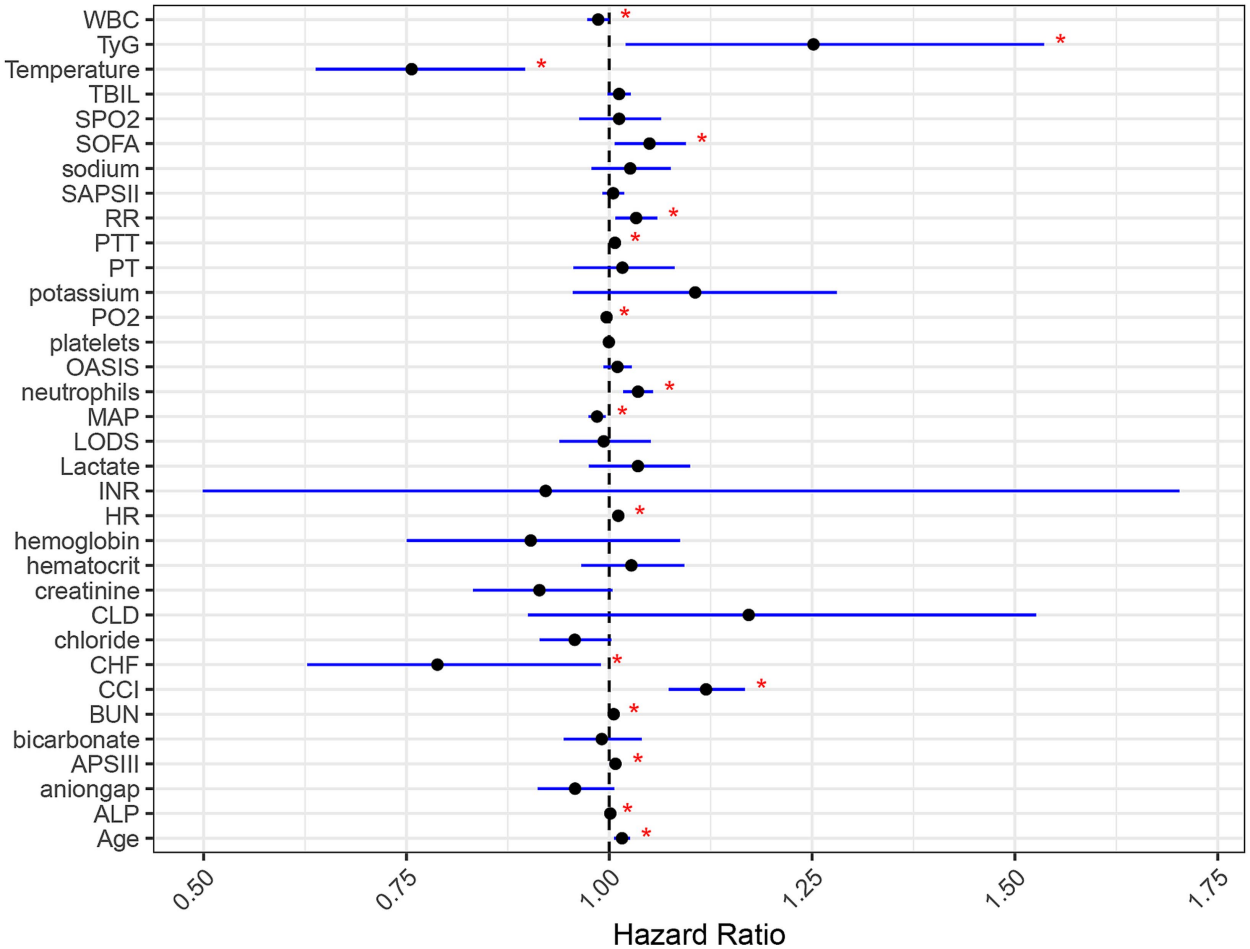
Forest plot of multifactorial cox regression analysis influencing the primary outcomes of SAE patients. Black dots denote the HR point estimates for each variable. Horizontal lines show the 95% confidence intervals; a line that crosses the vertical “1” line indicates a non-significant result. Blue lines signify that the HR is statistically significant, with a *p*-value less than 0.05, as marked by red asterisks.

These findings underscore that both TyG index and age are critical determinants influencing the 90-day overall survival of SAE patients. TyG index, in particular, exerts a significant impact on survival outcomes, whether assessed independently or in conjunction with other factors. The multifactorial Cox regression analyses, as illustrated in the forest plot, affirm that TyG index is an independent prognostic factor in SAE, with high TyG index levels being indicative of a poorer prognosis. Furthermore, other variables such as the SOFA score, OASIS score, CCI score, APSII score, along with higher age, heart rate (HR), respiratory rate (RR), prothrombin time (PTT), and neutrophil counts, were identified as additional risk factors for an adverse prognosis (Figure 4).

### 3.4 Correlation of TyG index with prognostic secondary outcomes in SAE patients

**Figure 5:**
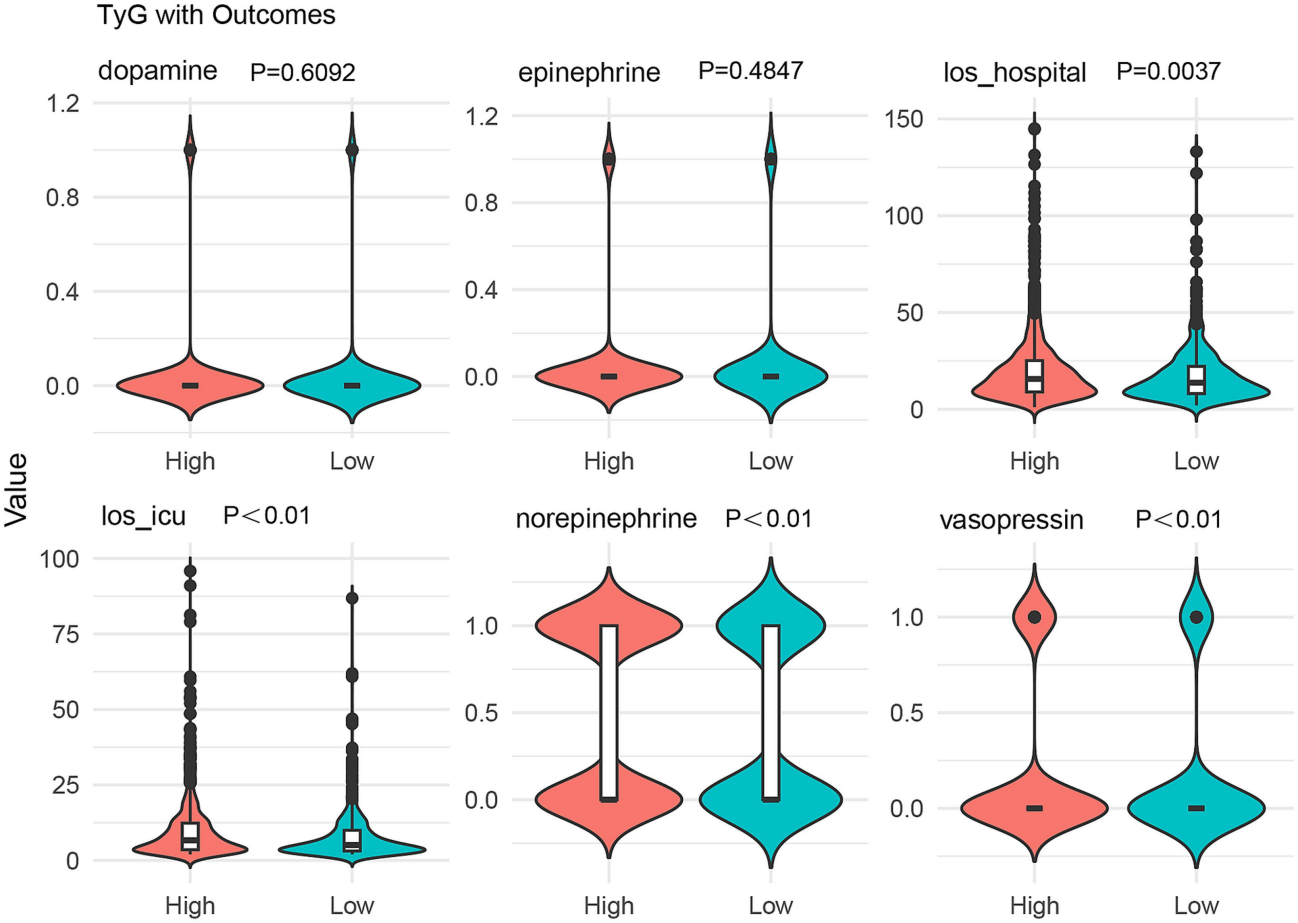
Violin plot showing the correlation between TyG index and secondary prognostic outcomes in SAE patients.

In this aspect of our study, we employed the Spearman rank correlation coefficient to assess the relationship of the TyG index with secondary prognostic outcomes in SAE patients. Our analysis revealed that the TyG index had a moderately positive correlation with the number of days spent in the Intensive Care Unit (ICU) (denoted as los_icu). This correlation emerged as statistically highly significant, with a *p*-value less than 0.01. In a similar vein, the correlation between the TyG index and the total number of days spent in the hospital, as well as the administration of norepinephrine and vasopressin, also demonstrated statistical significance (*p* < 0.01). Conversely, the association of the TyG index with the use of dopamine and epinephrine was found to be statistically insignificant and notably weak (Figure 5).

## 4 Discussion

In the present study, a comprehensive analysis was conducted on a cohort of 1,578 patients diagnosed with SAE. Among these patients, the median age was 65.96 years, with males being predominantly represented. This demographic profile aligns with the findings of Chen et al., where SAE patients had a median age of 67 years, with 57% being male (19). These consistent findings suggest that SAE predominantly affects an older demographic, with a slight male predominance. This observation may be indicative of the elevated susceptibility of the elderly to sepsis, and possibly, a higher susceptibility of men to serious infections (20). The current study affirms the TyG index as a significant predictor of disease severity and prognosis in SAE patients. Specifically, individuals in the high TyG index group exhibited elevated scores on the Clinical Criticality Score and more pronounced abnormalities in physiological parameters, including increased heart rate, respiratory rate, and body temperature. Moreover, the high TyG index group demonstrated a significantly heightened risk of mortality compared to the low TyG index group. These findings align with existing research; one study, for instance, established a relationship between SAE prognosis and sepsis severity, age, respiratory rate, body temperature, and heart rate (21). Another study revealed that SAE patients exhibited higher heart rate, blood lactate, and serum sodium levels, alongside lower platelet counts, serum albumin levels, and serum pH compared to non-SAE patients (22). Previous investigations have identified traditional severity scores, such as the Serial Organ Failure Assessment (SOFA) and the Acute Physiology and Chronic Health Evaluation (APACHE II), as important prognostic factors for SAE (19). In contrast to these studies, our findings suggest a relatively weak correlation between the TyG index and SOFA score. This disparity may stem from the diverse clinical dimensions assessed by the SOFA score (23), hinting at a more intricate interplay between various factors influencing SAE severity.

The TyG index serves as a biomarker reflecting systemic insulin resistance and lipid dysregulation, both of which are believed to contribute to the pathogenesis of serious adverse events (SAE) through multiple mechanistic pathways. First, hyperglycemia activates microglial cells via the AGE-RAGE axis, leading to an increase in blood–brain barrier (BBB) permeability, which subsequently promotes the infiltration of pro-inflammatory cytokines such as IL-6 and TNF-*α* into the brain parenchyma (24). Second, elevated triglyceride levels are hydrolyzed into free fatty acids (FFAs), which activate microglial cells through a TLR4-dependent NF-κB signaling pathway, exacerbating oxidative stress and mitochondrial dysfunction (25). Furthermore, insulin resistance inhibits the PI3K/Akt pathway, reducing glucose uptake by neurons and resulting in a collapse of energy metabolism (26). Collectively, these mechanisms form a “metabolism-inflammation-neuronal damage” vicious cycle, which may explain the strong association between the TyG index and poor prognosis in SAE.

Although the correlation between the TyG index and the SOFA score was relatively weak, the TyG index remains an important tool in assessing sepsis-associated encephalopathy (SAE). The severity of SAE is influenced by a variety of factors, involving complex interactions across metabolic, neurological, and inflammatory pathways. These intricate interactions suggest that a single metabolic marker, such as the TyG index, may not fully capture the comprehensive pathophysiological mechanisms of SAE. Nevertheless, as a simple, cost-effective, and reliable metabolic marker, the TyG index provides valuable insights into the metabolic status of patients, particularly in the early identification and risk prediction of SAE. Therefore, despite the weak correlation between the TyG index and the SOFA score, the role of the TyG index in evaluating the metabolic context of SAE and as a potential biomarker should not be underestimated. The TyG index may be a useful indicator of the metabolic disturbances associated with SAE. Future studies should investigate the combined use of the TyG index with other biomarkers to enable a more comprehensive assessment of SAE severity.

SAE, characterized by diffuse cerebral dysfunction resulting from a systemic inflammatory response, is primarily diagnosed based on the manifestation of impaired consciousness or delirium. Currently, this diagnosis is primarily one of exclusion. Numerous screening tools are available to identify delirium, yet none are specifically tailored to SAE. In a multicenter study, the CAM-ICU exhibited a sensitivity of 47%, specificity of 98%, and positive and negative predictive values of 95 and 72%, respectively (27). Consequently, the most suitable delirium screening tool for ICU settings remains a matter of debate. The ICDSC, with a higher sensitivity (99%) but lower specificity (64%) for delirium assessment compared to the CAM-ICU, presents an alternative option (28). The application of coma scales in SAE remains unexplored, although the GCS score proves useful in predicting the course of SAE (29). Neuroimaging findings in SAE patients exhibit variability, with acute abnormalities observed on MRI in a subset of cases, such as multiple ischemic strokes or hemianopic central white matter lesions (30). Notably, some patients exhibit normal brain MRI scans despite the presence of SAE.

SAE currently lacks specific biomarkers; hence, the identification of early warning and diagnostic indicators for SAE in critically ill septic patients holds significant importance for timely intervention and treatment (31). The underlying mechanisms through which the TyG index predicts SAE disease severity and the risk of death remain unclear. This study endeavors to explore this association from a pathophysiological perspective. The TyG index, serving as an indicator of metabolic disorders, is likely intricately linked to the onset of sepsis and SAE. Insulin resistance, a key component of metabolic disorders, not only affects systemic metabolism but may also impact hemodynamics and brain metabolism (32). Insulin resistance could contribute to blood–brain barrier dysfunction, increasing the brain tissue’s susceptibility to inflammatory responses and oxidative stress (33). These factors may exacerbate the symptoms and severity of SAE. Moreover, elevated levels of blood glucose and triglycerides may directly harm nerve cells, triggering the excessive release of inflammatory factors and cytokines (34). This, in turn, exacerbates the pathological progression of SAE. Such pathological changes may culminate in nerve cell death, further worsening neurological dysfunction.

Our study has several limitations that warrant consideration. Retrospective analyses based on a single database may introduce selection bias, thereby limiting the generalizability of our findings. Furthermore, while the TyG index proves valuable, it should be viewed as one component in the comprehensive assessment of patients with SAE, considering other relevant clinical parameters. Given the multitude of diseases that can lead to TyG alterations, its utility lies in identifying SAE, yet its specificity and sensitivity may be insufficient to distinguish SAE from other forms of encephalopathies. Nonetheless, owing to its ease of accessibility and lack of involvement of subjective factors, the TyG index holds importance in the early identification of SAE and monitoring treatment effects throughout the course of care.

In conclusion, our study underscores the significance of the TyG index as a vital independent predictor of disease severity and prognosis in individuals with SAE. The observed association with a poorer clinical prognosis and an elevated risk of mortality suggests its potential applicability in clinical practice. Future investigations should seek to validate these findings through prospective cohorts and delve into the underlying pathophysiological mechanisms of these associations. Such endeavors will contribute to advancing our comprehension of SAE, potentially paving the way for more effective management strategies for this challenging disease.

## 5 Conclusion

The TyG index is a significant independent predictor of disease severity and prognosis in SAE patients. High TyG levels correlate with worse clinical outcomes and increased mortality risk, suggesting its potential as a valuable tool in managing SAE.

## Data Availability

The datasets presented in this study can be found in online repositories. The names of the repository/repositories and accession number(s) can be found at: https://mimic-iv.mit.edu/.
